# Husbands’ involvement in family planning use and its associated factors in pastoralist communities of Afar, Ethiopia

**DOI:** 10.1186/s12978-019-0697-6

**Published:** 2019-03-18

**Authors:** Mebrahtu Kalayu Chekole, Znabu Hadush Kahsay, Araya Abrha Medhanyie, Mussie Alemayehu Gebreslassie, Afework Mulugeta Bezabh

**Affiliations:** 1 Department of Public health, college of health sciences, Samara University, Samara, Russia; 2 School of Public health, college of health sciences, Mekelle University, Mekelle, Ethiopia

**Keywords:** Afar, Family planning, Contraceptive, Afambo district, Pastoralist community, Husbands’ involvement

## Abstract

**Background:**

Husbands play an influential role in women’s access to health care, such as family planning services. However, there is little evidence of the level of husbands’ involvement in family planning services among pastoralist communities, who possess a distinct lifestyle. This study was aimed to assess husbands’ involvement in family planning use and factors associated in pastoralist communities of Afar, Ethiopia.

**Methods:**

Community-based cross-sectional survey was conducted among randomly selected 418 married women in Afambo district, Afar pastoralist community in 2017. Data were collected using semi-structured questionnaire. Data were entered to EPI-Info version 7 statistical software programs and exported to SPSS. Descriptive and multivariable logistic regression analyses were applied to identify factors associated with husband involvement. Odds ratio at 95% confidence interval were reported and significant association of factors was declared at the *p*-value of less than 0.05.

**Result:**

Four hundred eighteen married women were included in the study, making a response rate of 98%. The magnitude of husbands’ involvement in family planning was found to be 42.2%. Women who ever used family planning (AOR: 7.21; 95%CI: 3.58–14.67), those who participated in community networks, those who reported health center as their source of information for family planning (AOR: 5.56; 95%CI: 1.92–16. o7) were higher likely to report husband involvement compare to their counterparts. Participants’ increased knowledge was also significantly associated with higher odds of husband involvement in family knowledge (AOR = 1.31; 95% CI: 1.16–1.58). .

**Conclusion:**

Husbands’ involvement in the district is low. Women’s engagement in community networks aimed at increasing the knowledge of the women may involve in family planning. In addition, due focus among health care providers in lower health care units to provide information for both women and men might have a promising power to improve husbands involvement.

## Plan summary

Husbands’ involvement in family planning in study area is about 42.2% in the study area. The other thing is there a culture in the community everything is on the hand of the husband which is hierarchal, so no woman can decided independently. This hierarchal decision way is come from their ancestors and followed by every clan if not the woman may accused she is not obliged to the rule of the community or the clan. In addition to there are also other different reasons like the community is pastoralist and there is no access to basic health services and family planning and they lack information to all kind of reproductive health services due to they are far away from the basic infrastructure facilities. As recommendation mobile health is very crucial to attain the need of the community and husband education regarding family planning is another key to address the gap.

## Background

Male involvement in family planning refers to all organizational activities aimed at men as a discrete group which has the objective of increasing the acceptability and prevalence of the family-planning (FP) practice of’ either sex. This concept envisioned to address the prevalent women’s unmet need for family planning attributed to partner’s objection [1, 2]. Some of the reasons to involve men in FP activities and services rounds around the patriarchal orientations like male are the breadwinners, have access to information, fertile for a longer period of life, involved in polygamous relationships in most families and are the decision makers at all levels [3].

Therefore, male involvement in family planning has recently been understood as an important area among reproductive health program designers, policymakers, and population researchers [4].

Family planning uptake is low in pastoralist communities of Ethiopia [5–7] and other East African countries [2]. Literature also shows that women from pastoralist communities’ have limited capacity and autonomy in decision-making for family planning use, and substantial women missed to use it following fear of husband’s disapproval [8]. The factors that influence family planning in settled communities of Ethiopia are identified. Studies show that lack of awareness, religious prohibition, fear of side effect, think that family planning is the issue of women and desire of more children affects husband involvement in family planning use [9–12]. However, studies in the regard are scarce in pastoralist communities of Ethiopia. Federal Minister of Health (FMoH) Ethiopia planned also recognizes male engagement in family planning as a strategy to increase the prevalence of contraceptive rate [13]. Pastoralist community in Ethiopia accounts for 11% of the population and 5% of women in the reproductive age group [14].

Low in pastoralist communities, the decision on household matters, including fertility related issues belongs to the husband, which makes husbands’ involvement a critical issue [15, 16]. However, the level of the husband’s involvement is not determined while the factors that influence it are not well investigated in the pastoralist community setting. Understanding the magnitude of the husband’s involvement in the pastoralist setting will pave the strategies and policy recommendations to promote family planning. Therefore, the current study aimed to determine the level of the husband’s involvement in family planning use among married women from pastoralist women of Afar, Ethiopia.

## Methods and participants

### Study setting

Administratively, Afar region is divided into 5 zones and 34woredas, with a total population of 1.8 million as projected for the year 20,116. There were six hospitals, 58 health centers, and 294 health posts which are owned by the regional government in 2016 [17]. Afambo is one of the districts found in Zone 1 which is 150 km away from Samara town. In the district, there are 7 cables, and it has a total of 6449 women of reproductive age, 2 health centers and 11 health posts, and 30 Health Extension Workers (HEWs). The study was carried out from April 2017 to June 2017.

### Study design

A community-based cross-sectional survey design was conducted among married women to determine the magnitude of husband involvment in family planning and its factors associated. All married women aged 15–49 years who were eligible for the study.

### Sample size determination and sampling procedure

The sample size was calculated using Open-Epi 2.3, based on the following assumptions 80% power 95% CI, with a prevalence of 58.5% of Married Male who had information about contraceptive methods, odds ratio 2.5 [18], the design effect of 2 and 10% of non-response rate. Therefore, the final sample size was 418 married women. A multi-stage method was used to approach the study participants. The district has 7 administrative Kebeles. From the seven kebele, three Kebeles were randomly selected; namely Migo, Alasabolo and Humeduyta. Updated sanple frame was optained from local health extension workers in the kebeles and sample size was allocated to each kebele accordingly. Systematic random sampling method was applied to select study participants. Face to face interviews were conducted at women’s house. If two or more married women found in one household lottery method was applied.

### Data collection

The tool was developed in English after reviewing literature [7] and based on results found on qualitative exploration. A language expert translated it into the local language (Amharic). Data were collected using structured and pre-tested questionnaire. The tool consists of 1) socio-demographic characteristics 2) Knowledge, Attitude and practice of family planning 3) male involvement. Ten diploma female nurses and two supervisors, who were fluent in both Amharic and Afar Aff (local language) collected the data after they took 3 days of training.

To measure participant’s knowledge, participants were asked ten knowledge items in “Yes/No” form and the score of the participants for all knowledge items were summed up to compute the compasite of Knowledge score. Then, score was used for further analysis and treated as a continuous variable. Women were asked a “Yes /No” question if their husband involves either in giving advice on family planning use, or suggesting it, or accompany them to use family planning.

### Data analysis

Data were entered into EPI-Info version 7 statistical software programmes and finally exported to SPSS version 20.0 software for analysis. Summary statistics (mean, median and frequency) were made to summarize the characteristics of participants. Cross-tabulation (frequency with percentage) to show results of categorical variables while the mean (SD) was used to represent continuous variables after checking for normality of the distribution. In Bivariate logistic regression, each explanatory variable was assessed for significant association with the outcome variable. Finally, variables that were found significant in the bivariate logistic regression at the *p*-value of 0.25 were fitted to Multivariable logistic regression. The odds ratio at 5% confidence interval was reported and statistical significance of the association was declared at a P - value less than 0.05.

## Results

Four hundred eighteen married women were included in the study with a response rate of 98%. The mean age of the participants was 27.5 (±. 67), with a minimum age of 15 and a maximum of 49 years. The majority of them were Muslim (100%) and Afar in ethnicity (92%) and not attended formal school (85.4%. Three hundred thirty-four (81.5%) of the respondents’ pastoralist in occupation single wife to their husband 356 (86.8%) and resides in a rural area 276 (67.3%).

The average time traveling from home to near health institution was 37.24 min with SD 48 min; above half 226 (55.1%) of respondents were traveling less than 15 min, 58 (14.2%) traveled from 16 to 30 min and 126 (30.7%) of them traveled greater than 30 min. (Table 1).

**Table 1:** Socio-demographic characteristics of the study participants in the pastoralist community of Afambo district Afar, North Eastern Ethiopia, 2017 (*N* = 375)

Variables	Categories	Number (percentage/%)
Age	15–19	28 (7.4%)
	20–24	94 (25%)
	25–29	103 (27.5%)
	30–34	92 (24.5%)
	35–39	29 (7.7%)
	40–49	29 (7.7%)
Educational Status of women	Unable to read and write	319 (85.1%)
	Able to read and write	56 (14.9%)
Occupation of women	Pastoralist	307 (81.8%)
	Housewife	36 (9.6%)
	Employed	32 (8.5%)
Monogamy	Yes	323 (86.2%)
	No	52 (13.8%)
Residency	Rural community settlement	251 (66.9%)
	Move from place to place	124 (33.1%)
Distance from the health facility	<=15 min	222 (59.2%)
	16–30 min	55 (14.6%)
	> = 30 min	98 (26.2%)

### Women’s knowledge towards and practice on family planning

Almost all 96.6% of the participants have ever heard about family planning. Injectable (93%) and pills (83%) were frequently mentioned. Participants’ major source of information where health care providers 334 (81.5%) and HEWs (32%).

Participants mean score for ten knowledge items was 5.36 with SD of ±2. 527. Nearly one in three (29.5%) of the participants’ ever used family planning only 16.1% of were current family planning users. Moreover, 9 % of them did not disclose to their husband (Table 2).

**Table 2:** Family planning use among women from Afar pastoralist community of Ethiopia, 2017

Variables	Response	Number (percentage/%)
Source of information	Health Extension workers	306 (82%)
	Other health workers	69 (18%)
Ever use FP	Yes	121 (29.5%)
	No	289 (70.5%)
Current use of FP	Yes	66 (16.1%)
	No	273 (66.6%)
	Pregnant	71 (17%)
Type of FP methods	Injectable	61 (92.4%)
	Pills	5 (7.6%)
Source of family planning for users	Health center	52 (78.8%)
	Health post	8 (12.1%)
	Private health facility	5 (7.6%)
Motivate by	Self	35 (53%)
	Husband	31 (47%)

The proportion of women reported that their husband involves in advice, suggesting them or accompanying them for family planning was 42.2%. Almost half (49.3%) reported a history of husband objection to using family planning. The majority of the ever users (71.2%) reported that the decision to use family planning was done by both, while 21% reported by self. Only 7.6% of the users reported husband as decision maker (Table 3).

**Table 3:** Husbands’ involvement in family planning use in Afar pastoralist community of Ethiopia, 2017

Variables	Response	Non-users	Users
		N (%)	N (%)
Husband involvement			
Discussion about FP with your couple	Yes	96 (27.9%)	54 (81.8%)
	No	248 (72.1%)	12 (18.2%)
Discussion about spacing birth	Yes	73 (21.2%)	56 (84.8%)
	No	271 (78.8%)	10 (15.2%)
Discussion about limiting birth	Yes	48 (14%)	23 (34.8%)
	No	296 (86%)	43 (65.2%)
Husbands’ support among users			
Accompany me to the health facility for FP	Yes		24 (36.4%)
	No		42 (63.6%)
Participate in making the choice of type of FP	Yes		26 (39.4%)
	No		40 (60.6%)
Allow me to use for family planning	Yes		56 (84.8%)
	No		10 (15.2%)
Remind me of the schedule	Yes		40 (60.6%)
	No		26 (39.4%)
Help in me domestic activity	Yes		33 (50%)
	No		33 (50%)
Sharing me in use of family planning (use a condom)	Yes		0.00
	No		66 (100%)
Provide me financial support	Yes		52 (78.8%)
	No		14 (21.2%)
Aware of the side effect of the family planning	Yes		27 (40.9%)
	No		39 (59.1%)

### Factors associated with husband involvement in family planning use

Source of FP were health center, ever used by FP, knowledge and community participation were statistically significant predictors of husbands’ involvement in family planning use.

Family planning ever user women have been 7 times higher likely to report their husband’s involvement to compare to non- users (AOR = 7.2; 95%CI: 3.58–14.67). On the other hand, women who knew where family planning is found in health center reported that their husbands were 5 times (AOR: 5.5; 95%CI: 1.92–16.07) more likely involved in family planning utilization than their counterparts. Women who have a membership of 1 to 5 networks were 26% more their husbands’ involvement in family planning issue than their counterpart.

The study revealed that a 0.306 unit increase in women knowledge score had 1. 3 times (AOR = 1.3; 95% CI: 1.16–1.58) higher odds of husband’s involvement in family planning use after other variables in the model was adjusted (see Table 4).

**Table 4:** Factors associated with husbands’ involvement in family planning use in the afar pastoralist community of Ethiopia

Variables	Male involvement Family planning Use		COR	AOR (95% C.I.)	*P*-value
	Yes (173)	No (202)			
Ever use family planning					
Yes	86 (79.6%)	22 (20.4%)	8 (4.74,13.79)	7.2 (3.58,14.67)	0.000***
No	87 (32.6%)	180 (67.4%)	1	1	
Community participation					
Religious leader	39 (73.6%)	14 (26.4%)	3 (1.54,5.84)	3.6 (1.54,8.65)	0.003**
Clan leader	2 (20%)	8 (80%)	4.3 (0.89,20.75)	3.1 (0.52,18.45)	0.21
Health committee	8 (33.3%)	16 (66.7%)	0.53 (0.22,1.31)	1.9 (0.41,9.34)	0.39
1 to 5 net work	14 (19.4%)	58 (80.6%)	0.26 (0.13,0.49)	0.23 (0.09,0.58)	0.002**
No participation	104 (48.1%)	112 (51.9%)	1	1	
Monogamy					
Yes	157 (48.6%)	166 (51.4%)	6.3 (1.4,26.7)	0.58 (0.23,1.46)	0.25
No	16 (30.8%)	36 (69.2%)	1	1	
Residency					
Community settlement	129 (51.4%)	122 (48.6%)	3.08 (1.5,6.3)	1.3 (0.71,2.62)	0.34
Movable	44 (35.5%)	80 (64.5%)	1	1	
Knowledge	5.36 mean ± 2.53 SD	ß = 0.306	2.4 (1.4,4.1)	1.3 (1.16,1.58)	0.000***
Source of FP					
Health post					
Yes	125 (51.4%)	118 (48.6%)	1.7 (1.09,2.75)	1.09 (0.50,2.22)	0.88
No	41 (38%)	67 (62%)	1	1	
Hospital					
Yes	69 (66.3%)	35 (33.7%)	3 (1.88,4.92)	1.08 (0.50,2.32)	0.83
No	97 (39.3%)	150 (60.7%)	1	1	
Health center					
Yes	157 (54.1%)	133 (45.9%)	6.8 (3.24,14.35)	5.5 (1.92,16.07)	0.002**
No	9 (14.8%)	52 (85.2%)	1	1	
HCP as a source of information					
Yes	154 (50.2%)	153 (49.8%)	2.5 (1.46,4.61)	0.7 (0.28,1.84)	0.50**
No	19 (27.9%)	49 (72.1%)	1	1	

NB: HCP = health care provider, *P*-value < 0.0001 ***, < 0.05 **

## Discussion

This study aimed to assess the level of husband involvement in FP use and its associated factors among married reproductive age women in Afambo district of Afar, Ethiopia.

The overall magnitude of husbands’ involvement in Afambo district was found to be 42.2% in the study area. This finding was in line with a study done among married male in Gedo Town (40.8%) [18] But different from a study done in Amhara region, at Debre-Markos and Bahir-Dar (8.4, 25.5%), this discrepancy may be due to the time difference in these study and the socio-cultural difference of the community [12, 19]. However, the magnitude was higher in the study done in a Semi-Urban area of South -West region of Cameroon (57.2%) and this could be due to differences in the study setting [20].

Women who knew where family planning is found in health center were 5.5 times more likely to be associated with husbands’ involvement in family planning use than their counterparts. This is in line with a study done in southeast Nigeria to identify the Impact of male partner’s awareness and support for contraceptives on female intent to use contraceptives which showed that husbands’ who were aware the contraceptive of their wives’ where to be found were associated with their involvement [21]. This implies those basic infrastructures (health institution) are important for further improvement of husbands’ involvement and it indicates that exposure is crucial one and the catchment health facility will solve further with community settlement and/or mobile clinic to reach the hard to area.

The odds of male involvement among women their husbands’ were religious leaders was less than by 30% compared to those whose husband was not religious leader; and either the women or their husbands’ who have had participation in the community like one to five network were 26% more likely to involved their husbands’ in the issue of family planning positively. This might be due to there was myth and misconception among religious leaders that they believe the religion prohibited the use of family planning and leads to a negative effect. On the other hand, those who have community participation affected positively might be due to more exposure to health care providers and HEWs as they are the primary contact of the community and political assigned. It implies that the community needs to create awareness religion not prohibited to use family planning and make a different class to expose more to health education concern family planning like health development army.

The odds of husband involvement in family planning were found to be 8 times among women who were ever used of family planning compared to those who didn’t. This is similar with findings of the previous study conducted among married male at Gedo Town West Shoa Zone, Oromia, Ethiopia and in Debremarkos town, Northwest Ethiopia [12, 18]. This indicates that may be due to users have had good knowledge, because knowledge was correlated with current use of family planning (knowledge to current use = 0.58).

This study revealed that as the knowledge score of the women increased by 0.306 units the involvement of husband in family planning utilization was increased by 1.3 units. This is similar to a study done in Jimma zone and semi-urban areas of Cameroon [20, 22]. This indicates that the knowledge of the women could be a factor to husband involvement in family planning utilization and this implies family planning programs should be considered increasing the knowledge of the women by advocacy and awareness creation about family planning and the importance of husband involvement in family planning use.

## Conclusion

The study revealed that the magnitude of husbands’ involvement in family planning was 42.2%. It shows that there is a low level of husband involved in family planning use.

The independent factors associated with husbands’ involvement in family planning use knew health center as a source of family planning, ever used of family planning, knowledge of women on family planning use and community participation in either the woman or her husband.

## Recommendations

Strengthen the existing HEWs program, supportive and continuous follow up; HEWs are from the main source of information for FP and most reliable to them because they live with them and part of the community. Furthermore, HEWs can be the way to utilize the service for the community by the advocacy of the benefits of FP. Use the local influential leaders to increase husband involvement in FP, like religious and community leaders to influence husbands’ directly and indirectly, and one to five networks and health development army are also a good way to increase husbands’ involvement in family planning, so organize such team will be good. Community settlement to increase health institutions, accessibility and which is easy to reach the community.

## Abbreviations

AOR: Adjusted Odds Ratio
EDHS: Ethiopian Demographic Health Survey
FMoH: Federal Ministry of Health
FP: Family Planning
HEWs: Health Extension Workers
MPH: Masters of Public Health
RH: Reproductive Health
RIF: Reproductive health Innovation Fund
SD: Standard Deviation
SPSS: Statistical Package for Social Sciences
